# Plasminogen activator inhibitor-1 reduces cardiac fibrosis and promotes M2 macrophage polarization in inflammatory cardiomyopathy

**DOI:** 10.1007/s00395-020-00840-w

**Published:** 2021-01-11

**Authors:** Christian Baumeier, Felicitas Escher, Ganna Aleshcheva, Heiko Pietsch, Heinz-Peter Schultheiss

**Affiliations:** 1 Institute of Cardiac Diagnostics and Therapy, IKDT GmbH, Moltkestr. 31, 12203 Berlin, Germany; 2 Department of Cardiology, Charité University Medicine Berlin, Campus Virchow-Klinikum, Berlin, Germany; 3 German Centre for Cardiovascular Research (DZHK), Partner Site Berlin, Berlin, Germany

**Keywords:** Alpha-smooth muscle actin, DCM, DCMi, Inflammatory cardiomyopathy, Myocarditis, Cardiac fibrosis, Myofibroblast, Macrophages, Monocytes, PAI-1, TGF-β

## Abstract

Plasminogen activator inhibitor-1 (PAI-1) has a cardioprotective function in mice by repressing cardiac fibrosis through TGF-β and plasminogen-mediated pathways. In addition it is known to be involved in the recruitment and polarization of monocytes/macrophages towards a M2 phenotype in cancer. Here, we investigated the expression of PAI-1 in human dilated cardiomyopathy (DCM) and inflammatory dilated cardiomyopathy (DCMi) and its effect on cardiac fibrosis and macrophage polarization. We retrospectively analyzed endomyocardial biopsies (EMBs) of patients with DCM or DCMi for PAI-1 expression by immunohistochemistry. Furthermore, EMBs were evaluated for the content of fibrotic tissue, number of activated myofibroblasts, TGF-β expression, as well as for M1 and M2 macrophages. Patients with high-grade DCMi (DCMi-high, CD3^+^ lymphocytes > 30 cells/mm^2^) had significantly increased PAI-1 levels compared to DCM and low-grade DCMi patients (DCMi-low, CD3^+^ lymphocytes = 14–30 cells/mm^2^) (15.5 ± 0.4% vs. 1.0 ± 0.1% and 4.0 ± 0.1%, *p* ≤ 0.001). Elevated PAI-1 expression in DCMi-high subjects was associated with a diminished degree of cardiac fibrosis, decreased levels of TGF-β and reduced number of myofibroblasts. In addition, DCMi-high patients revealed an increased proportion of non-classical M2 macrophages towards classical M1 macrophages, indicating M2 macrophage-favoring properties of PAI-1 in inflammatory cardiomyopathies. Our findings give evidence that elevated expression of cardiac PAI-1 in subjects with high-grade DCMi suppresses fibrosis by inhibiting TGF-β and myofibroblast activation. Moreover, our data indicate that PAI-1 is involved in the polarization of M2 macrophages in the heart. Thus, PAI-1 could serve as a potential prognostic biomarker and as a possible therapeutic target in inflammatory cardiomyopathies.

**Supplementary Information:**

The online version contains supplementary material available at 10.1007/s00395-020-00840-w.

## Introduction

Dilated cardiomyopathy (DCM) is a non-ischemic disease of the heart muscle characterized by specific structural and functional abnormalities of the cardiac tissue [36]. It is clinically defined by left or biventricular dilatation and systolic dysfunction (LVEF ≤ 45%) in the absence of coronary artery disease, hypertension, valvular disease or congenital heart disease leading to heart failure and even sudden cardiac death [31, 35]. When DCM is accompanied by immune cell infiltration caused by infections and autoimmunity, it is defined as inflammatory dilated cardiomyopathy (DCMi) [29, 36]. DCM and DCMi are both often attended with fibrosis, increasing its morbidity and mortality [33]. Cardiac fibrosis is characterized by fibroblastic activation and fibrogenesis which in turn leads to elevated collagen fiber synthesis, altered myocardium architecture and potentiated risk of heart failure [21].

Plasminogen activator inhibitor type-1 (PAI-1, gene: *SERPINE1*) is a serine protease inhibitor that acts on the plasminogen system by inhibiting urokinase-type plasminogen activator (uPA) and tissue-type plasminogen activator (tPA). It is expressed in a variety of cell types and known to play an essential role in the progression of tissue fibrosis by regulating processes such as wound healing, matrix remodeling and immune cell migration. While genetic PAI-1 deficiency attenuates fibrosis in kidney [34], lung [20] and liver [2], lack of PAI-1 promotes spontaneous age-dependent fibrosis in mice heart [16, 40]. In agreement, a recent study reported spontaneous cardiac fibrosis in two young and healthy subjects with PAI-1 deficiency due to homozygous frameshift mutation in the *SERPINE1* gene [14].

The cardioprotective and anti-fibrotic effect of PAI-1 has been linked to improved endothelial to mesenchymal transition, enhanced activity of matrix metalloproteinases, increased vascular permeability, plasminogen inhibition and altered transforming growth factor-β (TGF-β) content [14, 16, 18, 40]. The latter is known to strongly contribute to fibrogenesis, as TGF-β induces fibroblast to myofibroblast transition [9, 22, 37]. Firstly, TGF-β is established to regulate PAI-1 expression [8, 28, 39], whereby novel findings demonstrate an upstream regulatory mechanism for PAI-1 in TGF-β mediated cardiac fibrosis [14, 19]. Supportingly, elevated plasma TGF-β level has been detected in two PAI-1 deficient subjects [14]. However, whether cardiac PAI-1 is involved in fibroblastic inhibition in human heart is unproven yet.

During myocardial injury, monocytes are recruited sequentially to sites of inflammation as part of the host-protective immune response. As a consequence, monocytes and macrophages make up to 75% of all infiltrating cells during myocarditis [4]. These bone marrow derived monocytes can differentiate to classical pro-inflammatory CD14^+^ CD68^+^ macrophages (M1 macrophages) or to non-classical immunosuppressive CD16^+^ CD68^+^ macrophages (M2 macrophages). This process is highly dependent on the cardiac microenvironment including cytokine and chemokine levels, inflammatory milieu as well as fibroblast presence [23]. Interestingly, PAI-1 has been suggested to be involved in the regulation of monocyte differentiation in cancer, as it promotes the recruitment and polarization of macrophages towards a M2 phenotype [26]. Thus, PAI-1 might be a crucial myocardial factor determining the fate of infiltrating monocytes during myocarditis and DCMi.

Here, we investigated the myocardial expression of PAI-1 in DCM and DCMi patients and examined its role in cardiac fibrosis and macrophage polarization.

## Materials and methods

### Patients

In total, 176 endomyocardial biopsies (EMBs) of patients (mean age 54.2 ± 15.2 years; 142 men) with the diagnosis of dilated cardiomyopathy (DCM, *n* = 27) or inflammatory dilated cardiomyopathy (DCMi, *n* = 149) were included in this retrospective study.

Coronary artery disease and other possible causes of myocardial dysfunction (hypertension, valvular heart disease) had been excluded by echocardiography and angiography prior to EMB in all patients. An ischemic genesis of DCM can therefore be ruled out. EMBs were obtained from the left or right ventricular septum using a flexible bioptome. Left ventricular ejection fraction (LVEF) was determined by echocardiography. DCMi patients were classified according to their CD3^+^ cell number into low-grade lymphocytic infiltration (DCMi-low, CD3^+^ lymphocytes = 14–30 cells/mm^2^, *n* = 88) and high-grade lymphocytic infiltration (DCMi-high, CD3^+^ lymphocytes > 30 cells/mm^2^, *n* = 61) (Table 1).

**Table 1:** Clinical characteristics and biopsy findings

Diagnosis	DCM	DCMi-low	DCMi-high
Number of patients, *n*	27	88	61
Men, *n* (%)	25 (93)	73 (83)	44 (72)
Age at diagnosis, Mean ± SD (years)	55.1 ± 15.3	54.5 ± 14.2	53.3 ± 16.7
LVEF at diagnosis, Mean ± SD (%)	27.5 ± 10.9	33.3 ± 14.1	25.0 ± 8.8^$$$^
PAI-1 level, Mean ± SEM (area %)	1.0 ± 0.1	4.0 ± 0.1	15.5 ± 0.4***^,$$$^
Inflammatory cell counts in biopsy			
CD3^+^ T-lymphocytes Mean ± SEM (cells/mm^2^)	7.8 ± 0.4	17.7 ± 0.1***	64.2 ± 0.9***^,$$$^
CD45R0^+^ T-memory cells Mean ± SEM (cells/mm^2^)	34.4 ± 0.4	67.1 ± 0.3***	130.6 ± 1.7***^,$$$^
LFA-1^+^ lymphocytes Mean ± SEM (cells/mm^2^)	11.0 ± 0.2	23.9 ± 0.1***	63.5 ± 0.9***^,$$$^
MAC-1^+^ macrophages Mean ± SEM (cells/mm^2^)	23.9 ± 0.4	52.0 ± 0.3***	103.0 ± 1.0***^,$$$^
Perforin^+^ cytotoxic cells Mean ± SEM (cells/mm^2^)	0.10 ± 0.01	1.15 ± 0.02**	4.12 ± 0.10**^,$$$^
ICAM-1^+^ cellular adhesion molecules Mean ± SEM (area %)	1.95 ± 0.03	3.18 ± 0.01***	4.26 ± 0.03***^,$$$^

**p ≤ 0.01, ***p ≤ 0.001 vs. DCM

^$$$^*p* ≤ 0.001 vs. DCMi-low

### Histology, immunohistochemistry and digital-imaging analysis

Histologic evaluation was performed on paraffin sections of two to three EMBs using standard procedures. In brief, formaldehyde-fixed biopsies were embedded in paraffin, cut into 4 µm thick slices and stained with Trichrome stain AB solution (Sigma, Taufkirchen, Germany), Bouin’s solution (Sigma, Taufkirchen, Germany) and Weigert A&B (Morphisto GmbH, Frankfurt, Germany). Quantification of fibrous connective tissue was performed using ImageJ 1.53A software. Active myocarditis was excluded according the histomorphologic Dallas criteria [1]. Immunohistochemistry was used to quantify inflammatory infiltrates and was carried out on RNAlater-fixed EMBs. Specimens were embedded in Shandon™ Cryomatrix (Thermo Fisher Scientific, Waltham, MA, USA), cut serially into cryosections of 5 µm thickness and placed on 10% poly‐L‐lysine‐precoated slides. Myocardial inflammation was assessed referring to the 2013 ESC statement [6]. In detail, inflammatory cardiomyopathy was diagnosed as CD3^+^ T-lymphocytes ≥ 14 cells/mm^2^ (1:700, Dako, Glostrup, Denmark), CD45R0^+^ T-memory cells ≥ 50 cells/mm^2^ (1:300, Dako, Glostrup, Denmark), CD11a^+^/LFA-1^+^ lymphocytes ≥ 14 cells/mm^2^ (1:250, Immuno Tools, Friesoythe, Germany), CD11b^+^/Mac-1^+^ macrophages ≥ 40 cells/mm^2^ (1:500, ImmunoTools, Friesoythe, Germany) and/or perforin^+^ cytotoxic cells ≥ 2.9 cells/mm^2^ (1:150, BD Bioscience, San Jose, CA, USA). Tissue and endothelial activation were measured by the expression of the intracellular adhesion molecule ICAM-1 (1:800, Immuno Tools, Friesoythe, Germany). As a secondary antibody, EnVision peroxidase‐conjugated anti‐mouse/anti-rabbit antibody (Dako, Glostrup, Denmark) was applied. Immunohistochemical staining was visualized using 3‐amino‐9‐ethylcarbazole as chromogenic substrate (Merck, Darmstadt, Germany). Finally, slides were counterstained in hematoxylin and mounted with Faramount aqueous mounting medium (Dako, Glostrup, Denmark). All markers were quantified using quantitative digital-imaging analysis as described before [11]. PAI-1 expression was analyzed on EMB cryosections using an appropriate antibody (1:1.000, Gentaur, Kampenhout, Belgium). PAI-1 expression area was quantified and depicted as percent of analyzed area (area fraction %). Quantification of myofibroblasts was performed on paraffin-embedded sections using an anti-alpha smooth muscle actin (α-SMA) antibody (1:200, Cell Signaling, Danvers, MA, USA). α-SMA^+^ cells were counted using ImageJ 1.53A software and are depicted as cells/mm^2^. TGF-β levels were quantified on paraffin-embedded sections using an anti-TGF-β antibody (1:1.000, antibodies-online GmbH, Aachen, Germany) and are depicted as percent of analyzed area (area fraction %).

### Macrophage differentiation

Quantification of classical M1 and non-classical M2 macrophages was conducted by immunohistochemical fluorescence staining. In brief, paraffin-embedded EMB sections were deparaffinized and incubated for 1 h at room temperature with mouse anti-CD68 antibody (1:80, Menarini, Florence, Italy) in combination with rabbit anti-CD14 (M1) and rabbit anti-CD16 (M2) antibodies (each 1:200, Cell signaling, Danvers, MA, USA), or rabbit anti-CD86 (M1) (1:50, Cell signaling, Danvers, MA, USA) and rabbit anti-CD163 (M2) antibodies (1:200, Medac GmbH, Wedel, Germany), respectively. Secondary antibodies were either anti-rabbit Alexa-488, or anti-mouse Alexa-546 labeled (Invitrogen, Carlsbad, CA, USA). Microscopy was performed using the confocal laser scanning microscope Leica-DMi8 (Leica Microsystems, Wetzlar, Germany). Las X 3.7.1.21655 software (Leica Microsystems, Wetzlar, Germany) was used to acquire and process images. Double positive cells (merge, yellow) were quantified using ImageJ 1.53A software.

### Quantitative real-time PCR

Total RNA extraction, cDNA synthesis, pre-amplification and gene expression analysis were performed as described previously [12]. *TGFB1* (Hs00998133_m1) mRNA levels were normalized to the expression of the housekeeping gene *HPRT1* (Hs99999909_m1).

### Statistical analysis

All data are displayed as mean ± SD or mean ± SEM, as indicated. For comparison of all groups, one-way analysis of variance (ANOVA) followed by Tukey’s multiple comparison test was used. All calculations were performed using GraphPad Prism version 6.00 (GraphPad Software, La Jolla, California, USA). Significance levels were set for *p*-values of less than 0.05 (*), 0.01 (**) and 0.001 (***).

## Results

### PAI-1 expression is elevated in DCMi patients with high-grade lymphocytic infiltration

**Fig. 1:**
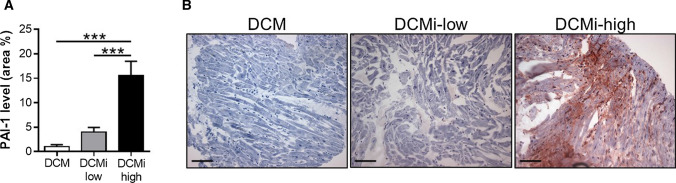
Cardiac PAI-1 expression is elevated in inflammatory-dilated cardiomyopathy with increasing lymphocytic infiltration. **a** Quantification of PAI-1 levels in endomyocardial biopsies of patients diagnosed for dilated cardiomyopathy (DCM) or inflammatory dilated cardiomyopathy (DCMi). DCMi patients were clustered into low-grade inflammation (DCMi-low, CD3^+^ lymphocytes = 14–30 cells/mm^2^) and high-grade inflammation (DCMi-high, CD3^+^ lymphocytes > 30 cells/mm^2^). Data are represented as mean ± SEM (*n* = 27–88 patients per group). ***p ≤ 0.001 as indicated. **b** Representative photomicrographs immunostained with anti PAI-1 antibody. Magnification: ×200. Scale bar: 100 µm

To elucidate the role of cardiac PAI-1 in dilated cardiomyopathies, we analyzed PAI-1 expression in EMBs of patients diagnosed for dilated cardiomyopathy (DCM, *n* = 27) or inflammatory dilated cardiomyopathy (DCMi) with either low-grade lymphocytic infiltration (DCMi-low, CD3^+^ lymphocytes = 14–30 cells/mm^2^, *n* = 88), or high-grade lymphocytic infiltration (DCMi-high, CD3^+^ lymphocytes > 30 cells/mm^2^, *n* = 61) (Table 1, Suppl. Figure 1). In DCM specimen, PAI-1 expression was negligible and not detectable in 26% of the samples (Fig. 1; 1.0 ± 0.1%), indicating a minor relevance of PAI-1 in the pathology of DCM. Patients with low-grade DCMi revealed a slightly, but not significantly elevated PAI-1 expression (Fig. 1; 4.0 ± 0.1%, *p* = 0.67). In contrast, PAI-1 was markedly increased in EMBs of subjects with high-grade DCMi (Fig. 1; 15.5 ± 0.4%, *p* ≤ 0.001), demonstrating a close connection between PAI-1 expression and the extent of inflammation in dilated cardiomyopathies. Interestingly, overall PAI-1 expression tend to be increased in female patients when compared to male counterparts (11.8 ± 3.6% vs. 6.5 ± 1.2%, *p* = 0.077).

### Increased PAI-1 in DCMi diminishes cardiac fibrosis by attenuating the activation of cardiac myofibroblasts

**Fig. 2:**
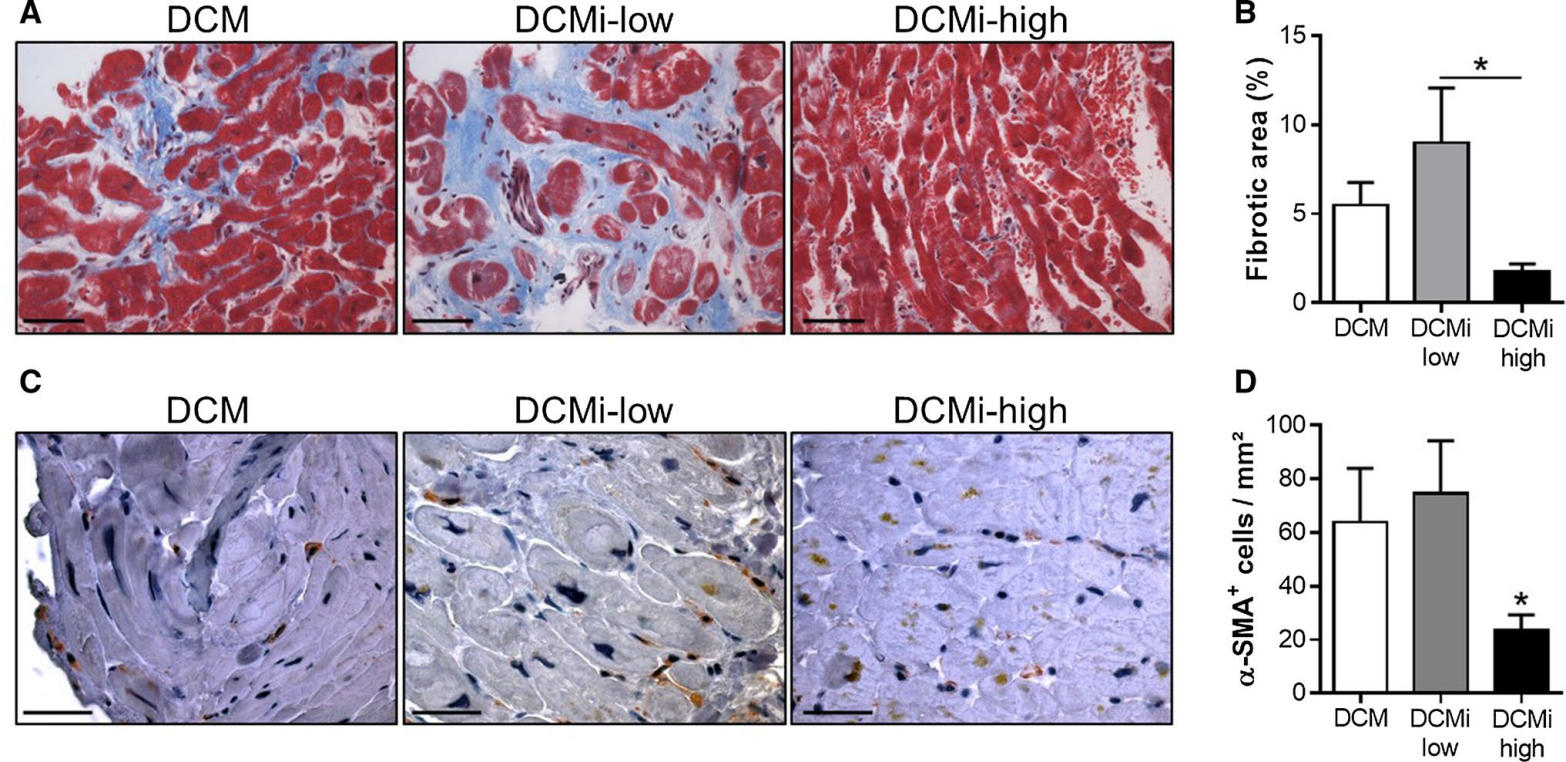
Increased PAI-1 level in DCMi-high patients attenuates fibrosis by inhibiting the activation of cardiac myofibroblasts. **a** Representative photomicrographs from cardiac sections of patients with DCM, DCMi low-grade inflammation (DCMi-low) and DCMi high-grade inflammation (DCMi-high) stained with Trichrome stain AB solution. Fibrous connective tissue is identified in blue. Magnification: ×400. Scale bar: 20 µm. **b** Quantification of fibrotic area (*n* = 6–9 patients per group). **c** Representative photomicrographs from cardiac sections immunostained with anti α-smooth muscle actin (α-SMA) antibody. α-SMA^+^ myofibroblasts are identified in brown. Magnification: ×600. Scale bar: 40 µm. **d** Quantification of α-SMA^+^ cells (*n* = 6–12 patients per group). Data are represented as mean ± SEM. *p ≤ 0.05 vs. DCM and DCMi-low or as indicated

PAI-1 is known to protect mice against cardiac fibrosis [16, 17, 25, 40]. However, whether PAI-1 suppresses fibrosis in human heart is not clear yet. Histological examinations on Trichrome-stained EMB sections show the highest fraction of fibrous connective tissue in DCMi-low patients, which was reduced by 80% in DCMi-high subjects (Fig. 2a, b). Supportingly, immunohistochemical staining of EMBs for activated myofibroblasts revealed a decreased prevalence of α-SMA^+^ cells in DCMi-high patients when compared to DCM and DCMi-low subjects (Fig. 2c, d; 32.2 ± 3.1 cells/mm^2^ vs. 63.8 ± 8.2 cells/mm^2^ and 70.1 ± 5.2 cells/mm^2^; *p* ≤ 0.05). Collectively, our data indicate that elevated PAI-1 expression in DCMi-high patients is correlated to lower number of extracellular matrix (ECM) producing myofibroblasts and consequently to a reduced expansion of fibrous connective tissue. Thus, PAI-1 exhibits anti-fibrotic effects in hearts of DCMi patients.

### Elevated TGF-β content in inflammatory dilative cardiomyopathy is attenuated by PAI-1

**Fig. 3:**
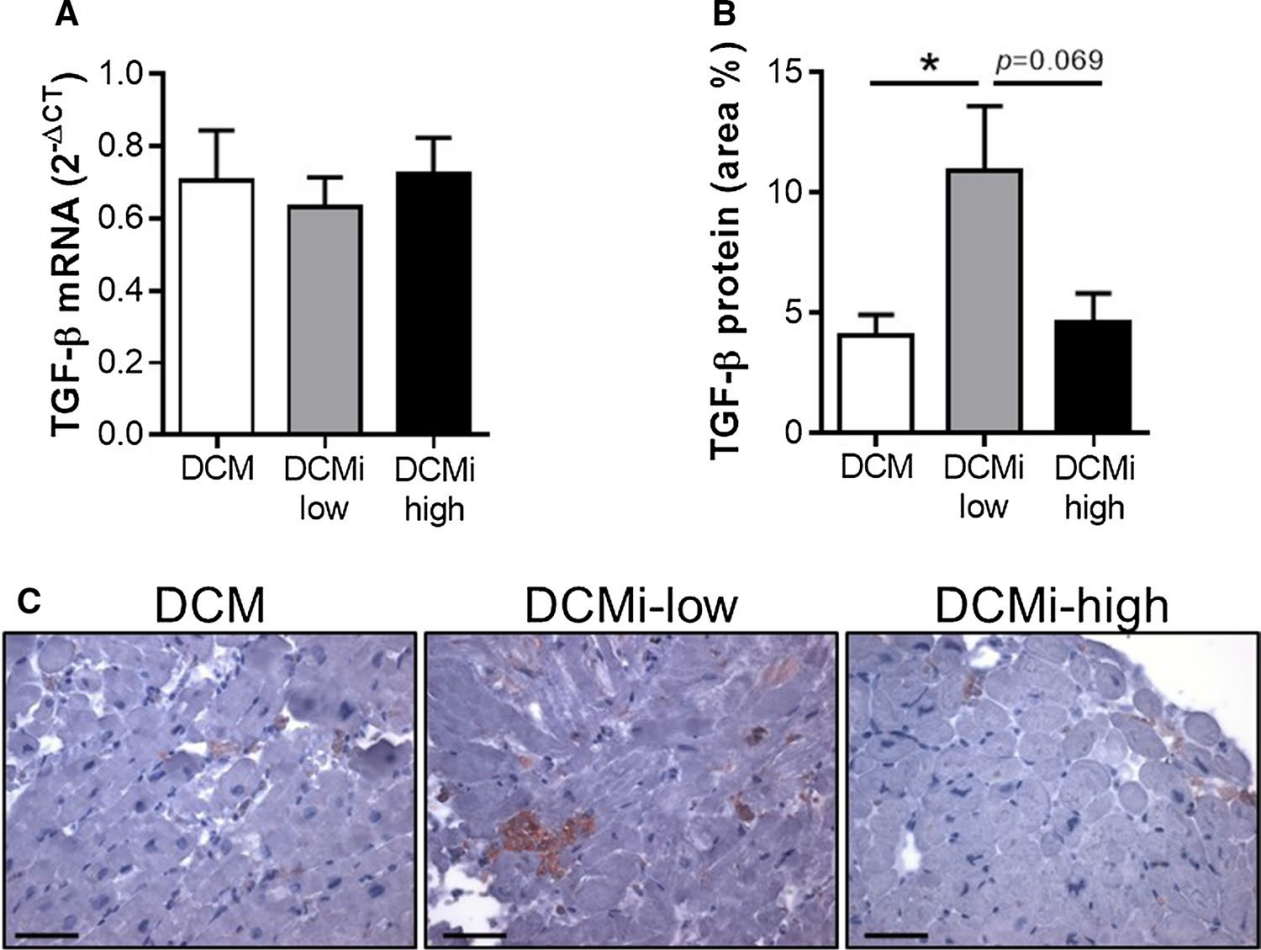
Increased expression of TGF-β in inflammatory dilative cardiomyopathy is attenuated by elevated PAI-1 levels. **a**, **b** Relative TGF-β mRNA (**a**) and protein (**b**) levels in endomyocardial biopsies of patients diagnosed for dilative cardiomyopathy (DCM) or inflammatory dilative cardiomyopathy (DCMi). DCMi group were clustered into low-grade inflammation (DCMi-low, CD3 + lymphocytes = 14–30/mm^2^) and high-grade inflammation (DCMi-high, CD3 + lymphocytes > 30/mm^2^). Data are represented as mean ± SEM (*n* = 7–12 patients per group). *p ≤ 0.05 as indicated. **c** Representative photomicrographs immunostained with anti-TGF-β antibody. Magnification: ×400. Scale bar: 20 µm

Since the anti-fibrotic effect of cardiac PAI-1 is presumably mediated via TGF-β, we next analyzed cardiac TGF-β expression on mRNA and protein levels. Interestingly, despite *TGFB1* mRNA levels were not affected by PAI-1 (Fig. 3a), TGF-β protein content was increased in DCMi-low patients, which was diminished by tendency in DCMi-high subjects (Fig. 3b, c; 10.9 ± 2.7% vs. 4.6 ± 1.2%, *p* = 0.069). Thus, these findings provide further evidence of an involvement of PAI-1 in the regulation of cardiac fibrosis by inhibiting TGF-β on a post-translational level.

### PAI-1 promotes macrophage polarization towards a M2 phenotype

**Fig. 4:**
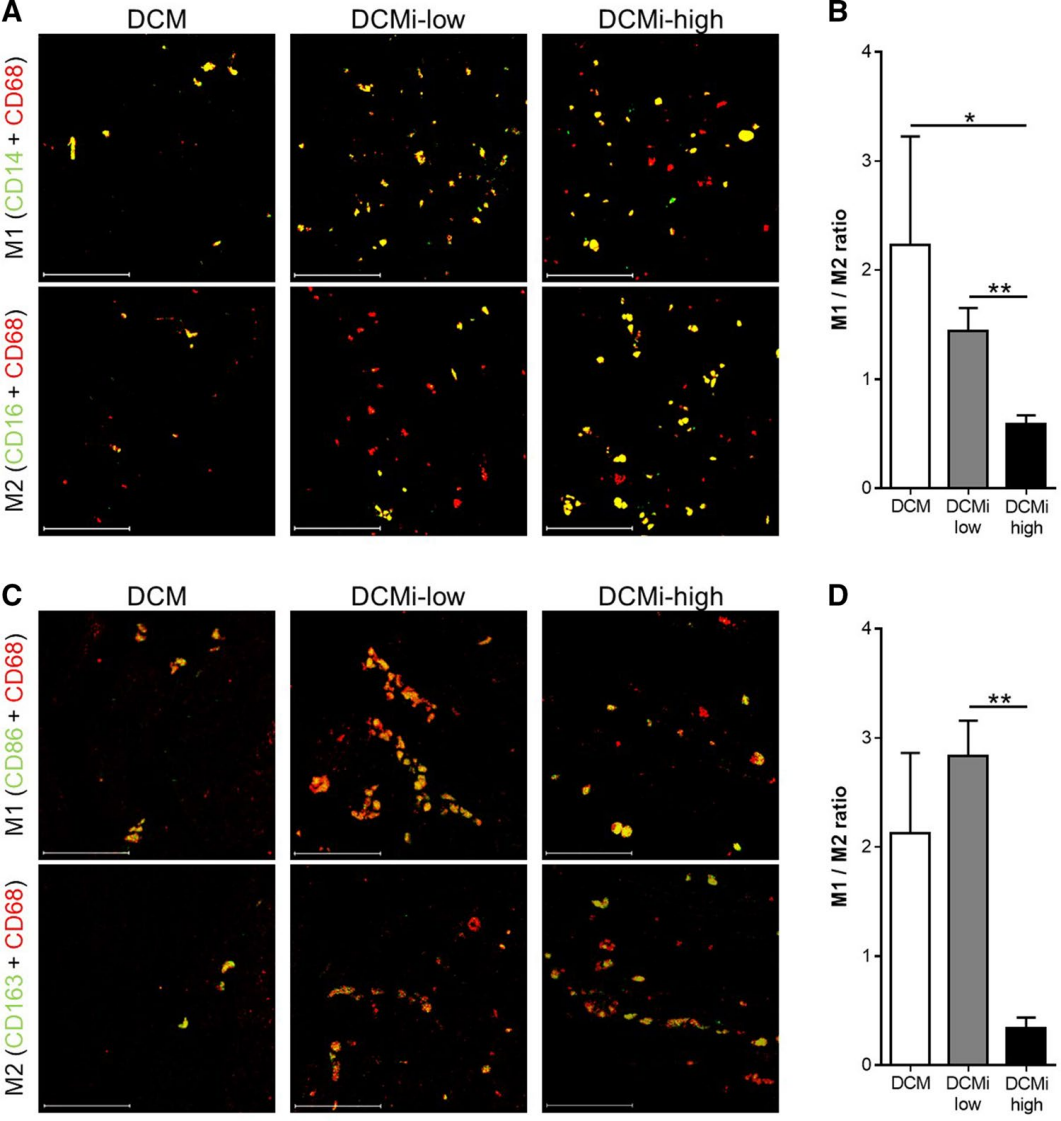
Cardiac PAI-1 expression is associated with increased prevalence of M2 macrophages. **a**, **c** Representative photomicrographs from cardiac sections of patients with DCM, DCMi low-grade inflammation (DCMi-low) and DCMi-high grade inflammation (DCMi-high), double immunostained against CD14/CD68 (green/red) and CD16/CD68 (green/red) (**a**), or CD86/CD68 (green/red) and CD163/CD68 (green/red) (**c**), respectively. Double positive cells are depicted in yellow and indicate presence of M1 (CD68^+^ CD14^+^ and CD68^+^ CD86^+^) or M2 (CD68^+^ CD16^+^ and CD68^+^ CD163^+^) macrophages. Magnification: ×400. Scale bar: 50 µm. **b**, **d** M1 to M2 ratio was calculated from the total number of M1 (CD68^+^ CD14^+^ (**b**) or CD68^+^ CD86^+^ (**d**) double positive cells) and M2 (CD68^+^ CD16^+^ (**b**) or CD68^+^ CD163^+^ (**d**) double positive cells) macrophages (*n* = 6–11 patients per group). Data are represented as mean ± SEM. *p ≤ 0.05, **p ≤ 0.01

It was recently shown in vitro, that PAI-1 promotes the recruitment and polarization of M2 macrophages through its uPA interactive domain in cancer [26]. However, the role of cardiac PAI-1 in macrophage differentiation is still illusive. Compared to DCM, total number of MAC-1^+^ macrophages was twice as much in EMBs of the DCMi-low group (23.9 ± 0.4 vs. 52.0 ± 0.3 cells/mm^2^, *p* ≤ 0.001) and even 4-times higher in DCMi-high patients (103.0 ± 1.0 cells/mm^2^, *p* ≤ 0.001) (Table 1, Suppl. Figure 1). Confocal fluorescence microscopic analysis of the prevalence of M1 (CD68^+^ CD14^+^) and M2 (CD68^+^ CD16^+^) macrophages revealed a M1/M2 macrophages ratio less than 1 in DCMi-high patients, pointing out a majority of M2 macrophages in these samples (Fig. 4a, b). In contrast, DCM and DCMi-low patients show a M1/M2 ratio greater than 1, indicating an excess of M1 compared to M2 macrophages (Fig. 4a, b). However, since the majority of CD68^+^ human cardiac macrophages was shown to be also CD14 positive [3], we applied additional markers to discriminate between M1 and M2 macrophages. In line with the initial analysis, double immunofluorescence staining of M1 (CD68^+^ CD86^+^) and M2 (CD68^+^ CD163^+^) macrophages show strongly reduced M1/M2 ratio in DCMi-high subjects when compared to DCMi-low patients (Fig. 4c, d). In summary, using various macrophage polarization markers, our data clearly link cardiac PAI-1 levels to monocyte polarization towards anti-inflammatory M2 macrophages.

## Discussion

The present data reveal that (i) subjects suffering from high-grade DCMi exhibit elevated cardiac PAI-1 expression compared to DCM and low-grade DCMi patients. Moreover, we show that (ii) increased PAI-1 levels in DCMi contribute to a reduced level of cardiac fibrosis by (iii) decreasing TGF-β expression and (iv) diminishing the number of myofibroblasts. Finally, we demonstrate that (v) PAI-1 leads to a shift of pro-inflammatory M1 macrophages towards anti-inflammatory M2 macrophages. This is to our knowledge, the first study demonstrating a direct effect of cardiac PAI-1 expression on myofibroblast activation and macrophage differentiation in human endomyocardial tissue.

The cardioprotective and anti-fibrotic effect of PAI-1 has been extensively studied using genetically modified mouse models [16, 19, 40]. Contrary to other organs such as liver, lung and kidney, PAI-1 deficiency and overexpression of its downstream target uPA promotes cardiac-selective fibrosis [32]. On the other hand, transgenic mice overexpressing human PAI-1 develop coronary artery thrombosis [10], and elevated PAI-1 is discussed to be a risk factor for thrombosis and atherosclerosis [38]. However, besides one report of two PAI-1 deficient subjects developing spontaneous cardiac fibrosis [14], evidence of PAI-1 mediated fibrogenesis in human heart is missing. Mouse studies show that PAI-1 represses cardiac fibrosis by inhibiting myocyte TGF-β synthesis [14, 19], whereas PAI-1 was shown to enhance TGF-β expression in human macrophages [42]. As TGF-β is known to activate myofibroblasts, it is likely that PAI-1 reduces fibrotic activity via the inhibition of TGF-β mediated myofibroblast activation. Myofibroblasts are the activated form of fibroblasts that express elevated levels of α-SMA and display a markedly enhanced ability to produce ECM. Here, we demonstrate that compared to the disease picture of DCM, cardiac TGF-β content was induced by inflammatory events in DCMi-low patients, which was completely abolished in DCMi-high subjects exhibiting elevated PAI-1 levels. Interestingly, *TGFB1* mRNA expression was not different between the groups, suggesting a regulatory mechanism of PAI-1 on a post-translational level, as it was proposed previously [19]. The reduced TGF-β level in DCMi-high subjects in turn led to a diminished number of activated α-SMA^+^ myofibroblasts, suggesting a lower capacity of ECM synthesis in these patients. Indeed, histological examination revealed a decreased presence of fibrous connective tissue in DCMi-high patients. However, considering the endocard-close origin of the analyzed EMBs, absolute quantification of fibrosis in these biopsies is problematic. Factors such as wall tension, chronicity, hemodynamics and disease progression make the evaluation of fibrotic events difficult. Thus, data on fibrotic area need to be interpreted carefully. Nevertheless, our data strongly support the current view of the anti-fibrotic capacity of cardiac PAI-1 and provide further evidence for its involvement in TGF-β regulation and myofibroblast activation in human heart.

We further demonstrate, that increased PAI-1 levels cause massively induced polarization of M2 (CD68^+^ CD16^+^) in relation to M1 (CD68^+^ CD14^+^) macrophages. Bone marrow derived circulating monocytes can differentiate into “classical” inflammatory (M1) and “non-classical” patrolling (M2) monocytes. The expression of the surface proteins CD14 and CD16 in combination with the pan-macrophage marker CD68 can be used to distinguish between these two populations [23]. However, as it was shown that the majority of heart resident human CD68^+^ macrophages co-express CD14 in dilated and ischemic cardiomyopathies [3], we applied CD86 and CD163 as additional markers for M1 and M2 macrophages, respectively [5]. Concurrent with the first analysis, DCMi-high patients revealed an excess of M2 (CD68^+^ CD163^+^) towards M1 (CD68^+^ CD86^+^) macrophages, underlining the effect of PAI-1 on macrophage polarization. In this regard, a dual function of PAI-1 on monocyte/macrophage migration and polarization has been recently suggested in cancer [26]. PAI-1 is involved in the coordination of Mac-1 dependent macrophage migration [7] and monocyte polarization [26] via p38MAPK and NF-κB dependent IL-6 and STAT3 activation [24, 26, 27]. As IL-6 and STAT3 activation promote macrophage polarization towards M2 phenotype in mice [13, 15, 30], it is likely that PAI-1 mediates M2 polarization via p38MAPK/NF-κB/IL-6 dependent signaling pathway. The observed high number of M2 macrophages in DCMi-high patients points toward a similar mechanism in human cardiac tissue. However, since M2 macrophages can contribute to angiogenesis, genesis of myofibroblasts and collagen deposition [41], reduced myofibroblast number and increased M2 macrophages might be contradictory and need to be evaluated in further studies.

In summary, our data provide further evidence for an inhibitory effect of PAI-1 on cardiac fibrosis and give an insight into its role in macrophage polarization in human heart. Moreover, our data link pronounced inflammatory dilated cardiomyopathy to enhanced PAI-1 levels, which might serve as an important marker for prognosis of fibrogenesis and in turn for mortality.

## Study limitation

There are limitations of a retrospective analysis which have to be considered when interpreting the obtained results. These include, among other factors, a lack of extended clinical data for all of the patients covered in this study. Moreover, the proportion of gender and disease pattern might differ due to distinct clinically suspected diagnosis and a usual excess of male patients. Finally, the presented data are rather correlative and proposed mechanisms need to be evaluated in further studies.

## Supplementary Information

Below is the link to the electronic supplementary material.

Supplementary file1 (DOCX 529 KB)
