# Supplementary material for: Plasminogen activator inhibitor-1 reduces cardiac fibrosis and promotes M2 macrophage polarization in inflammatory cardiomyopathy
Source: Basic Res Cardiol. 2021 Jan 11;116(1):1. doi: 10.1007/s00395-020-00840-w (PMC7801308; doi:10.1007/s00395-020-00840-w)

**Supplementary Figure 1:** Representative immunohistochemical images of patients diagnosed for dilative cardiomyopathy (DCM) or inflammatory dilative cardiomyopathy (DCMi), with either low grade inflammation (DCMi-low, CD3^+^ lymphocytes = 14-30 /mm²) or high grade inflammation (DCMi-high, CD3^+^ lymphocytes > 30 /mm²). (A) CD3^+^ T-lymphocytes, (B) CD45R0^+^ T-memory cells, (C) LFA-1^+^ lymphocytes, (D) MAC-1^+^ macrophages, (E) Perforin^+^ cytotoxic cells and (F) ICAM-1^+^ cellular adhesion molecules. Magnification: 400x. Scale bar: 20µm.


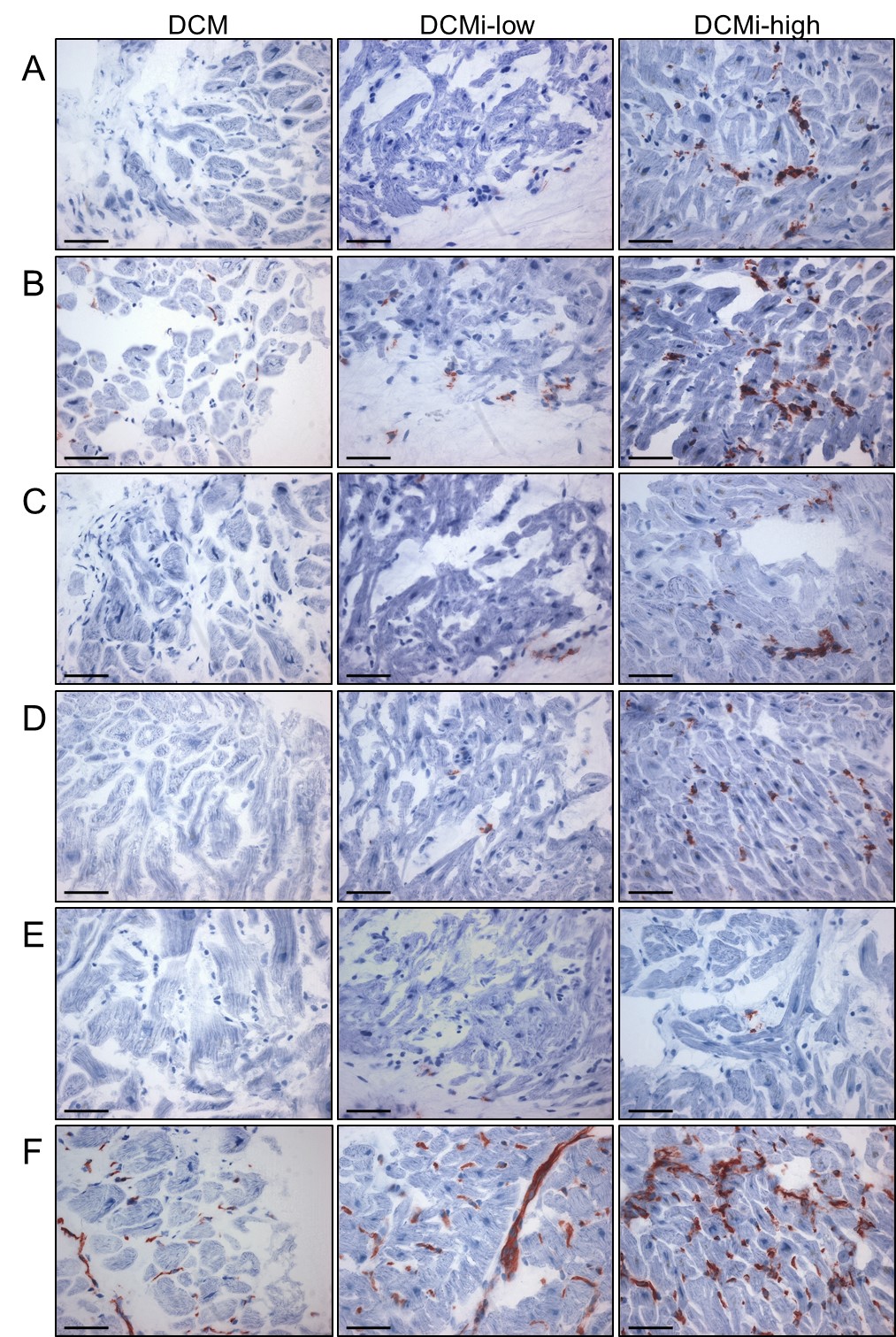

Supplement: Supplementary file 1 — Supplementary file1 (DOCX 529 KB) [file 395_2020_840_MOESM1_ESM.docx]
